# Spatial transcriptomics of retinoblastoma: a visual window on intra-patient heterogeneity

**DOI:** 10.1186/s12885-025-14814-5

**Published:** 2025-09-02

**Authors:** A. P. Moulin, J. Thevenet, L. Mazzeo, S. Tissot, C. Stathopoulos, F. L. Munier, A. Berger

**Affiliations:** 1 Fondation Asile des Aveugles, Jules-Gonin Eye Hospital, Lausanne University, Lausanne, Switzerland; 2 Department of Oncology, Immune Landscape Laboratory Platform, CHUV, Lausanne, Switzerland

**Keywords:** Retinoblastoma, Spatial transcriptomics, Molecular signature, Histological features, Retinocytoma.

## Abstract

**Background:**

Retinoblastoma is the most common intraocular malignant tumor in childhood. Although current treatments offer a high survival rate, treatment toxicity, tumor relapse, and treatment resistance require a deeper understanding of the disease mechanisms to develop adapted therapies. Microscopically, this tumor is characterized by different states of differentiation and proliferation, ranging from poorly differentiated to well-differentiated retinoblastoma. Retinocytoma, on the other hand, is a benign non-proliferative tumor. Recent next-generation multi-omics analyses classified retinoblastoma tumors in subtypes 1 or 2, subtype 2 presenting a later age of onset, more genetic alterations, and higher metastatic potential. In parallel, several single-cell transcriptomics studies demonstrated intratumoral heterogeneity. However, mapping the different cell populations directly on the tumor and comparing histological features and molecular subtypes remains an unmet need.

**Methods:**

Spatial transcriptomics was used to characterize a primary enucleated case with two histologically distinct areas. The whole transcriptomic profile of sixteen regions of interest, covering the two differentiation patterns of the tumor and the non-tumoral retina, was analyzed.

**Results:**

The clustering of the regions of interest based on whole transcriptome data correlated with the histological description: cluster 1 (6 regions of interest) corresponded to highly differentiated areas and cluster 2 (6 regions of interest) to poorly differentiated areas. They showed enrichment for phototransduction and proliferation respectively, confirmed by immunohistochemistry for markers of these pathways. The publicly available molecular signatures of the two retinoblastoma subtypes categorized our regions of interest into two groups, which correlated perfectly with histological observation and transcriptomic profiles. Further analysis of the expression of specific senescence markers in the well-differentiated area did not support the diagnosis of retinocytoma as it did not confirm the expected up-regulation seen in this tumor type.

**Conclusions:**

This study demonstrates a strong correlation between histological observation and molecular profiling, representing the first mapping of retinoblastoma composed of adjacent molecular subtypes 1 and 2. It also highlights the diagnostic ambiguity between retinocytoma and well-differentiated non-proliferative subtype 1 retinoblastoma and emphasizes the need for specific biomarkers to differentiate these two tumor types.

**Supplementary Information:**

The online version contains supplementary material available at 10.1186/s12885-025-14814-5.

## Background

Retinoblastoma affects 1/17’000 live births, representing the most common malignant intraocular tumor. In most cases, tumorigenesis is initiated by biallelic inactivation of the *RB1* gene encoding for pRB, originally described as the Knudson two-hit hypothesis [1]. Management of this tumor has considerably advanced over the past 20 years, especially with the development of intraarterial, intravitreal and, more recently, intracameral chemotherapy [2–4], allowing not only major improvements in patient survival but also increasing globe salvage. However, current treatment is associated with frequent anesthesia, potential treatment-related side effects, tumor relapse, and no guarantee of eye or vision preservation [5]. Additionally, metastatic disease remains incurable with conventional treatment leading to poor prognosis, especially in cases involving the central nervous system. A deeper molecular understanding of the disease is needed to improve retinoblastoma treatment, vision preservation, and overall patient quality of life.

Retinoblastoma is believed to arise during retinal development, in a cone precursor with a unique sensitivity to pRB loss [6]. The evaluation of human embryonic retinal explants has suggested that the initial proliferative state occurred during cone maturation, followed later by an indolent phase which might lead to either retinocytoma (benign tumor) or retinoblastoma (malignant tumor) development [6, 7]. Retinocytoma has been described histopathologically as a homogeneous population of well-differentiated cells with round nuclei containing bland speckled chromatin, variable degree of photoreceptors differentiation with numerous fleurettes, and absence of mitosis. Found in about 16% of enucleated retinoblastoma, retinocytoma is suspected to be in a senescent phase with increased p16 and p130 expression [7] but no molecular signatures have yet been made available. Recently, single-cell RNA sequencing (scRNA-seq) analysis from primary enucleated eyes identified several clusters of cells that could be subdivided into immature cone precursors and retinoblastoma cells [8, 9]. Pseudotime analysis suggested an evolution from a premalignant immature cone precursor into a retinocytoma or a retinoblastoma state. Others suggested cone precursors in the G2/M phase at the origin of retinoblastoma [10].

A recent multiomics study has led to a better understanding of the molecular landscape of retinoblastoma with the identification of 2 subtypes, subtype 1 occurring at an early age with very few mutations and preservation of late cone markers (e.g. ARR3), and subtype 2 occurring later, with a slightly increased mutation burden, aneuploidies, ganglion/neuronal (e.g. EBF3) and stemness genes expression, as well as an increased metastatic risk (with TFF1 as a proposed marker) [11]. In this study, the authors identified in 32% (8/25) of subtype 2 tumors a mutually exclusive pattern of expression of ARR3 and EBF3. In 3 of these cases, the ARR3+, non-proliferative (KI67-) areas were reminiscent of retinocytomas. ScRNA-seq and copy number variation analysis from one subtype 2 case with mutually exclusive ARR3 + and EBF3 + areas revealed the presence of 6 tumor cell populations, half with late cone markers and few aneuploidies, and the other half with ganglion cell markers and increased aneuploidies.

This heterogeneity and the possible co-existence of both retinoblastoma subtype 1 and 2 within the same tumor has been described several times, but never directly observed and mapped on a patient sample. The interpretation of the transcriptomic programs of two adjacent tumor types within the same retinoblastoma sample, in correlation with their histological description and characterization, will enhance our understanding of the disease’s molecular features. Herein, we present the use of spatial transcriptomics to map and delineate the transcriptomic programs of well and poorly-differentiated cell populations identified in a primary enucleated case.

## Methods

### Patient sample

The selected case of this study is a primary enucleated eye from a 3-year-old boy presenting a unilateral non-germline group E retinoblastoma.

### Spatial transcriptomics

FFPE tissue section of 5-µm is baked at 60 °C for 1 h. The slide is deparaffinized in 3 xylol baths of 5 min, then rehydrated in ethanol gradient from 100% ethanol, 2 baths of 5 min followed by 95% ethanol, 5 min. The slide is then washed in PBS 1X.

For RNA detection, antigen retrieval is performed in Tris-EDTA pH 9.0 buffer at 100 °C for 15 min at low pressure. The slide is first maintained in hot water for 10 s then deep into Tris-EDTA buffer. The cooker vent stays open during the procedure to ensure low pressure and reach 100 °C. The slide is then washed into PBS 1X, and incubated into proteinase K (1 μg/ml) in PBS for 15 min at 37 °C, and washed again in PBS 1X. Tissue is post-fixed in 10% neutral-buffered formalin 5 min, washed 2 times 5 min in NBF stop buffer (0.1 M Tris Base, 0.1 M Glycine) and finally one time in PBS 1X. The mix of Whole Transcriptomic Atlas probes (Nanostring Technologies) is dropped on each section and covered with HybriSlip Hybridization Covers. The slide is then put for hybridization overnight at 37 °C in a Hyb EZ II hybridization oven (Advanced cell Diagnostics). The HybriSlip cover is gently removed the day after and 25-min stringent washes are performed twice in 50% formamide and 2X SSC at 37 °C. Tissue is washed for 5 min in 2X SSC, then blocked in Buffer W (Nanostring Technologies) for 30 min at room temperature in a humidity chamber. Next, 500 nM Syto83 and antibodies targeting AF488-Synaptophysin (clone SY38, Merck), AF594-CD45 (clone 2D1, Bio-techne), and AF647-CD68 (clone KP1, Santa Cruz Biotechnology) in Buffer W are applied to section for 1 h at room temperature. The slide is washed twice in fresh 2X SSC then loaded on the GeoMx Digital Spatial Profiling (DSP).

For slide collection, the entire slide is imaged at ×20 magnification, and morphologic markers are used to select Region of Interest (ROI) either using circle or organic shapes. ROIs are exposed to 385 nm light (UV), releasing the indexing oligonucleotides which are collected with a microcapillary and deposited in a 96-well plate for subsequent processing. The indexing oligonucleotides are dried down overnight and resuspended in 10 µl of DEPC-treated water.

NGS sequencing platform is used as readout. Sequencing libraries are generated by PCR from the photo-released indexing oligos and ROI-specific Illumina adapter sequences, and unique i5 and i7 sample indices are added. Each PCR reaction used 4 µl of indexing oligonucleotides, 4 µl of indexing PCR primers, 2 µl of Nanostring 5X PCR Master Mix. Thermocycling conditions are 37 °C for 30 min, 50 °C for 10 min, 95 °C for 3 min; 18 cycles of 95 °C for 15 s, 65 °C for 1 min, 68 °C for 30 s; and 68 °C for 5 min. PCR reactions are pooled and purified twice using AMPure XP beads (Beckman Coulter, A63881), according to the manufacturer’s protocol. Pooled libraries are paired-sequenced at 2 × 27 base pairs and with the single-index workflow on an Illumina Novaseq instrument.

Novaseq-derived FASTQ files for each sample are compiled for each compartment using the bcl2fastq program of Illumina, and then demultiplexed and converted to digital count conversion files using the GeoMx DnD pipeline version 2.0.0.16 of NanoString according to manufacturer’s pipeline.

Quality control and Q3 normalization for RNA Dataset are performed using the R package GeoMxTools v3.4.0. Probes are checked for outlier status. A probe is removed if the geometric mean of that probe’s counts from all segments divided by the geometric mean of all probe counts representing the target from all segments is less than 0.1 or if the probe is an outlier according to the Grubb’s test in at least 20% of segments.

The counts for all remaining probes for a given target are then collapsed into a single metric by taking the geometric mean of probe counts.

Segments and genes are filtered out with abnormal low signal. Segments with less than 5% of gene, and genes with less than 5% of segments detected and below the limit of quantification set at 2 geometric standard deviation above the geometric mean, are removed from the study.

Gene counts are normalized to the geometric mean of the 75th percentile across all ROIs to give the upper quartile (Q3) normalization factors for each ROI.

### Immunohistochemistry

A deeper molecular characterization of this sample was performed with the detection of pRB (AbFrontier LF-MA0173, 1:300), KI67 (DAKO M7240, 1:300), ARR3 (Proteintech 11100-2-AP, 1:1000), GUCA1A (kindly provided by Prof. Alexander Dizhoor, 1:2000), GUCA1B (kindly provided by Prof. Alexander Dizhoor, 1:2000), CKAP2 (Sigma-Aldrich, 1:500), TOP2A (Sigma-Aldrich HPA006458, 1:400), KIFC1 (Sigma-Aldrich HPA055997, 1:3000) and TFF1 (Sigma-Aldrich HPA003425, 1:1000). All antibodies were incubated for 30 min at room temperature.

### Cell cycle prediction

Cell cycle was predicted using CellCycleScoring of Seurat. As previously described [9], the normalized scores of S phase and G2/M phase of each ROI were calculated by using the average expression levels of 43 S-phase marker genes and 54 G2/M-phase marker genes (Seurat package provided).

### Bioinformatic analysis

Differential gene expression (DEG) between groups of segments was assessed on Q3 normalized data using the Linear modeling feature of the limma package (version 3.58.1). Gene set enrichment analysis (GSEA) over the Hallmak gene sets Gene Ontology, Biological Process subset, from the Molecular Signature Database (MSigDB) were performed with the R package, ClusterProfiler (version 4.8.1). T-distributed Stochastic Neighbor Embedding (tSNE) was conducted using Rtsne (version 0.16). The visualization of specific gene expression on the image data was done using the R package SpatialOmicsOverlay (version: 1.2.1).

For heatmap generation, the pheatmap (version: 1.0.12) package was utilized. The data matrixes were provided to the pheatmap function along with clustering parameters, custom color palettes, and annotation information derived from an external annotation file.

Gene Set Variation Analysis (GSVA) (v1.50.2; R v4.3.2) was used to quantify enrichment of published retinoblastoma subtype signatures [11]. A filtered expression matrix (genes × ROIs, log₂(CPM + 1)) and two gene-set lists (“Subtype1”, “Subtype2”) were input to GSVA, which computes per‐sample enrichment scores. The two gene-set lists were built on published signatures (top 500 enriched genes in each subtype, data extracted from supplementary Table 3) [11]. Resulting scores were reshaped with tidyr (v1.3.1) and visualized as bar charts in ggplot2 (v3.5.0), grouped by gene set (shown as a heatmap, red for positive; blue for negative, for each subtype) and applying custom axis labels, titles, and margins.

ESTIMATE [12] (v1.0.13) was applied to infer tumor purity and stromal/immune content from the same log­normalized expression matrix. After exporting to GCT and filtering with filterCommonGenes(), scores were generated by estimateScore(), read back into R, and converted to tumor purity via the published cosine formula: TumorPurity = cos(0.6049872018 + 0.0001467884×ESTIMATEScore),

with percentages calculated as TumorPct = 100×TumorPurity and NonTumorPct = 100 – TumorPct. Tumor vs. non­tumor fractions per ROI were plotted side-by-side in ggplot2.

EPIC [13] deconvolution was used to resolve the non-tumor compartment into seven defined cell types (CAFs, endothelial cells, B cells, CD4 T cells, CD8 T cells, macrophages, NK cells). The log₂(CPM + 1) matrix was input to EPIC with withOtherCells = FALSE in order to look exclusively at the immune and stromal fractions; any ROIs failing convergence were re-fitted with rangeBasedOptim = TRUE. The final cellFractions matrix (16 × 7) was converted to percentages and plotted as stacked bars in ggplot2, using a fixed, publication-ready color scheme for each population.

## Results

### Transcriptomic profiling corresponds to histological features

**Fig. 1:**
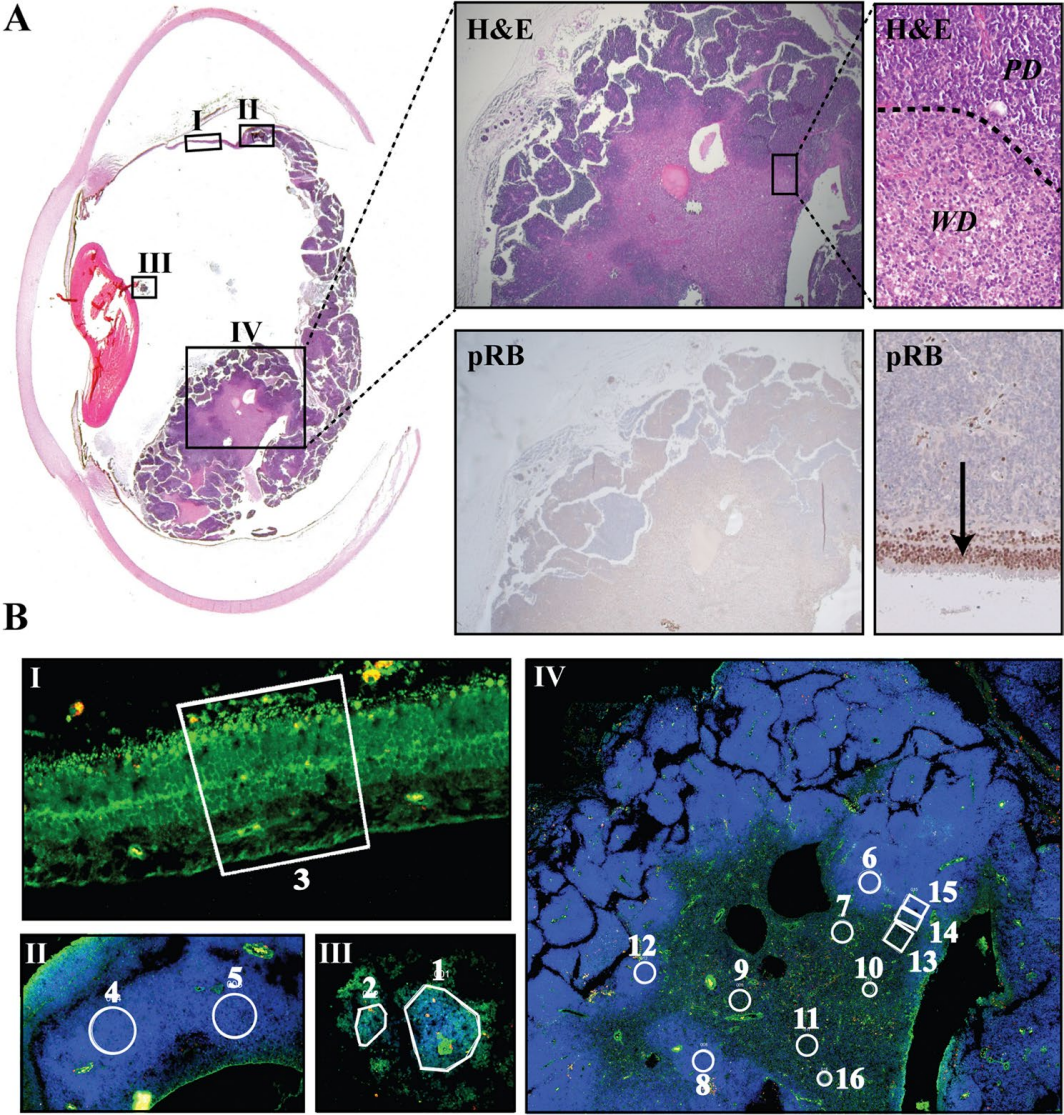
Histopathological features of the selected case and ROIs selection. **A **Left and top panels: Retinoblastoma with a mostly endophytic growth observed with Hematoxylin and Eosin (H&E) staining on FFPE (Formalin-fixed Paraffin-embedded) section of the full patient eye. The four rectangular areas (I to IV) correspond respectively to the retina, the distant intraretinal tumor, the vitreous seeds, and the central intraretinal tumor. The zoomed regions illustrate both the well-differentiated (WD) and poorly-differentiated (PD) retinoblastoma. Bottom panels: staining for pRB is negative in the tumor (*RB1* loss) but positive in the retina. The arrow shows the photoreceptor nucleus. **B** FFPE slide treated for GeoMx Digital Spatial Profiling (see methods). The areas lined by white borders correspond to the selected ROIs (Regions Of Interest). ROI # 3 was selected in normal retina; ROIs # 4 and 5 in distant poorly differentiated retinoblastoma; ROIs # 1 and 2 in poorly differentiated vitreous seeds; ROIs # 7, 9, 10, 11, 13 and 16 were selected in the well-differentiated area; ROIs 6, 8, 12, 15 in the poorly differentiated area, and ROI 14 at the interface between the well-differentiated and poorly-differentiated areas

Histopathological examination of a unilateral naïve non-germline group E retinoblastoma demonstrated a poorly differentiated (PD) tumor with mixed endophytic and exophytic growth, postlaminar optic nerve extension, along with vitreous and aqueous humor seeding with no choroidal invasion. Serial sections revealed a well-differentiated (WD) area with cells containing regular oval nuclei and numerous fleurettes, possibly reminiscent of a retinocytoma (Fig. 1A) as previously described [7]. Loss of RB expression was observed in both the WD and PD areas (Fig. 1A). Whole transcriptome analysis was performed in a total of 16 selected regions of interest (ROI) including non-tumoral retina in the periphery of the eye (area I, ROI #3), PD cells in vitreous seeds (area III, ROI #1, 2), in the periphery (area II, ROI #4, 5) and in the main tumor area (area IV, ROI #6, 8, 12, 15), WD cells (area IV, ROI #7, 9, 10, 11, 13, 16) as well as a transition zone (area IV, ROI #14) between the PD and WD areas (Fig. 1B).

**Fig. 2:**
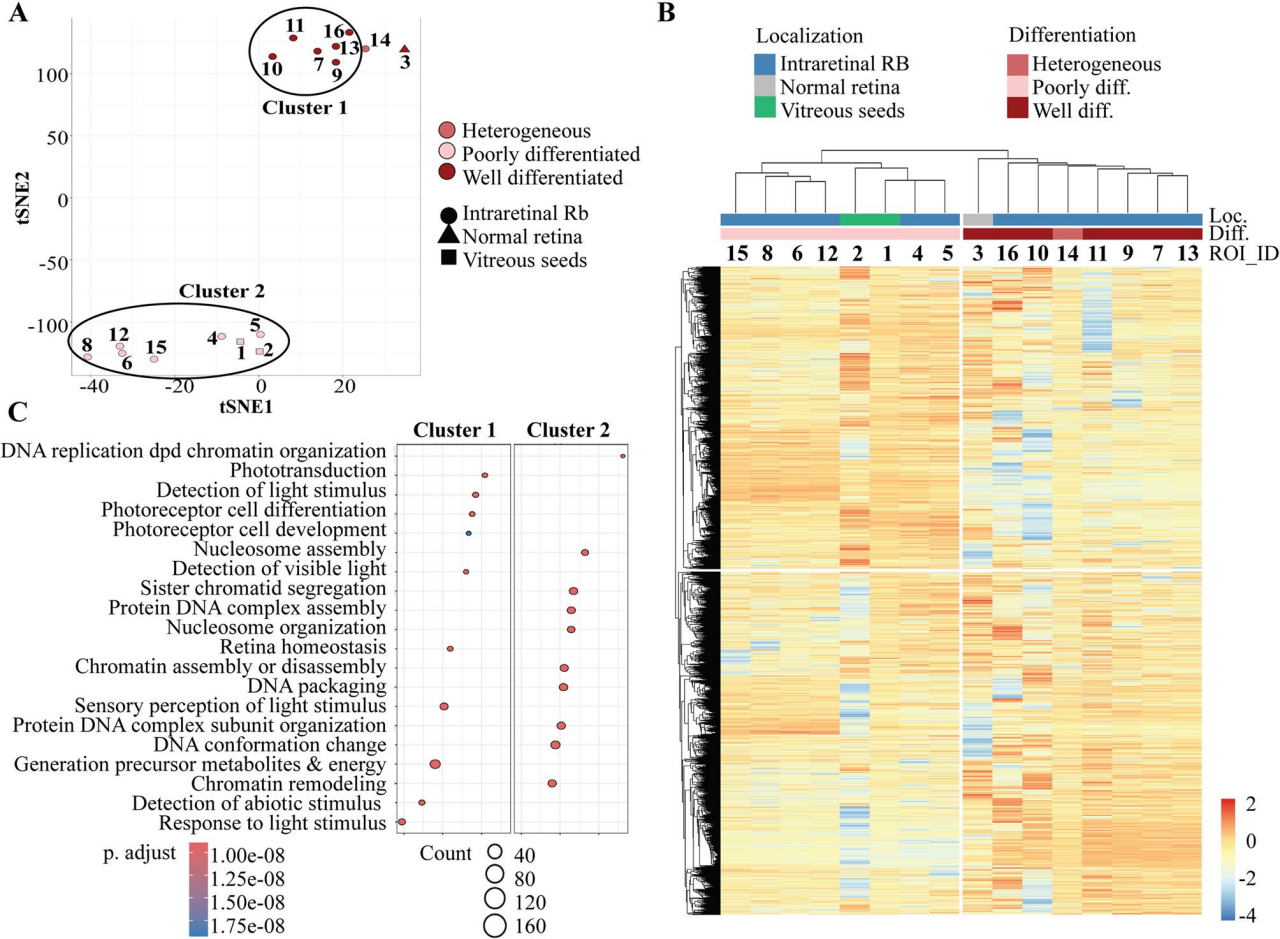
Transcriptional program of a heterogeneous retinoblastoma defined by spatial transcriptomics. **A** tSNE visualization of the 16 ROIs. **B** Unsupervised clustering of the 16 ROIs based on the 1988 deregulated genes in ROIs of cluster 1 versus cluster 2 identified in A. The expression level of each of the deregulated gene is represented in the heatmap. **C** Gene Set Enrichment Analysis showing the Gene Ontology – Biological Processes enriched in cluster 1 and 2 ROIs

The transcriptomic data, visualized by tSNE, generated two distinct clusters, perfectly correlating with the histological observations. Cluster 1 corresponded to all the WD regions (ROI #7, 9, 10, 11, 13, and 16) and was close to the non-tumoral retina. Cluster 2 corresponded to the integrality of the PD regions, independently of their localization in the primary tumor (periphery or center, ROI #4, 5, 6, 8, 12 and 15) (Fig. 2A). A total of 1998 genes were differentially expressed between the two clusters; whose expression level also grouped the ROI #3 and 14 with the 6 WD ROI (Fig. 2B).

### Lineage-specific versus proliferative cell populations

**Fig. 3:**
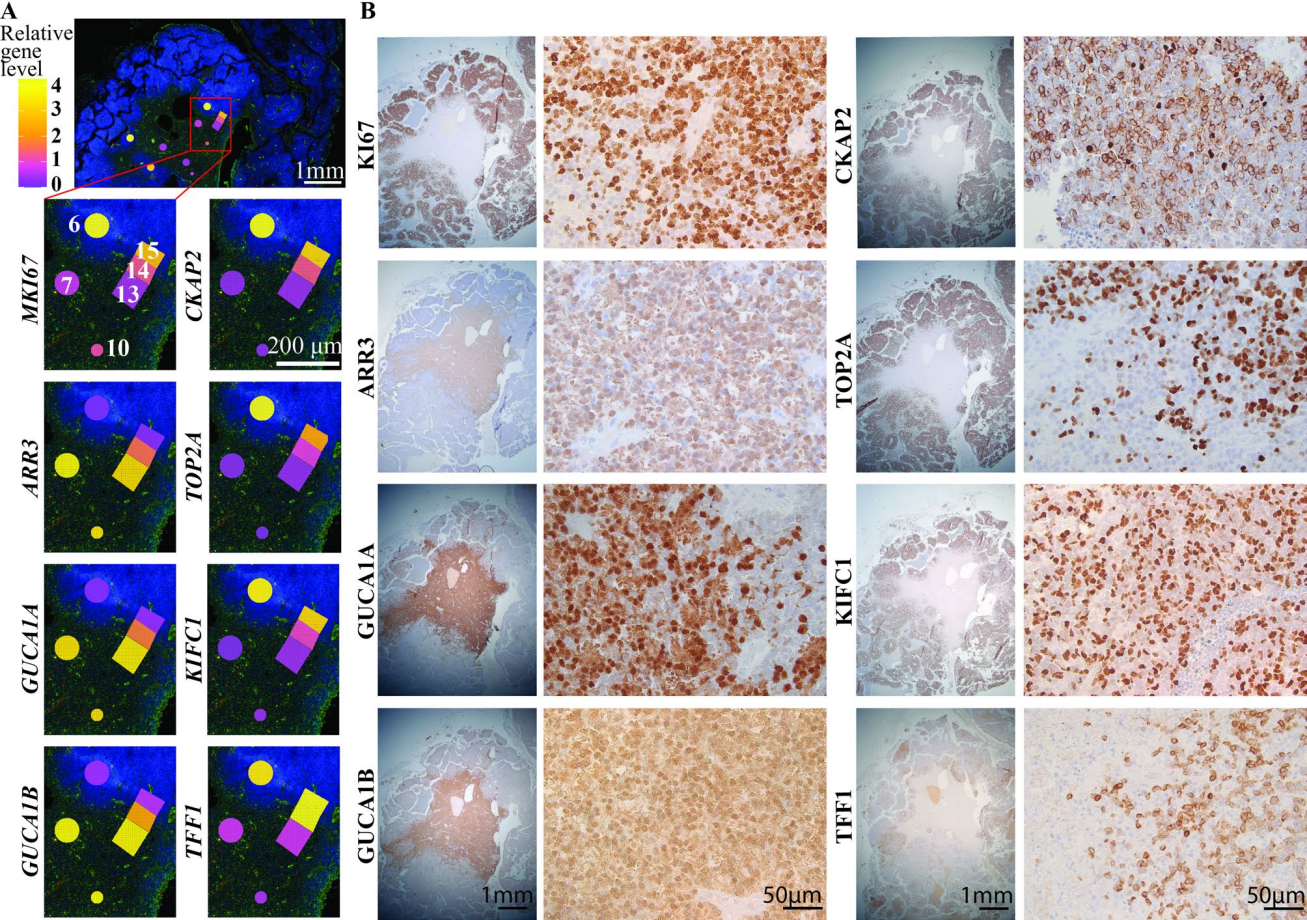
Visualization of expression level of top deregulated targets and biologically relevant markers. A Spatial reconstitution of the RNA level in ROIs # 6, 7, 10, 13, 14, and 15 of the indicated genes. B Immunohistochemistry of the corresponding targets. ARR3, GUCA1A, GUCA1B are expressed in the well-differentiated area while MKI67, CKAP2, TOP2A, and KIFC1 are expressed in the surrounding poorly-differentiated area. Heterogeneous expression of TFF1 in the poorly-differentiated area. Magnifications: x13 and x252

Gene set enrichment analysis (GSEA) of the deregulated genes revealed enrichment for biological processes such as phototransduction, photoreceptor cells, and response to light stimulus in cluster 1, consistent with a late cone lineage specificity, as previously described in subtype 1 retinoblastoma [11]. On the other hand, DNA packaging and replication, as well as chromatin remodeling were enriched in cluster 2, indicating a higher proliferative potential of this cell population (Fig. 2C), as observed in subtype 2 retinoblastoma [11]. The expression of some of the top differentially expressed genes can be visualized in WD ROIs (# 7, 10, 13), PD ROIs (# 6 and 15) and the transition ROI #14 (Fig. 3A). We confirmed at the protein level, by immunohistochemistry, the expression of mature cone markers in the WD areas (ARR3, GUCA1A, GUCA1B) and genes associated with cell division and microtubule organization (KIFC1, CKAP2) as well as DNA transcription and replication (TOP2A) in the adjacent PD areas. TFF1, a recently characterized marker of metastatic potential observed in subtype 2 retinoblastoma [11, 14], was also heterogeneously expressed in PD areas only (Fig. 3B). Regarding the tumor microenvironment, the relative balance between malignant and immune components in each ROI showed no significant difference in tumor purity between WD areas and PD areas (Additional Fig. 1A-B). A deeper analysis of immune and stromal compartments also indicated no significant differences between WD and PD areas for B cells, CD4 + T cells, CD8 + T cells, macrophages and NK cells (Additional Fig. 1C-D).

### Molecular subtypes of retinoblastoma versus retinocytoma

**Fig. 4:**
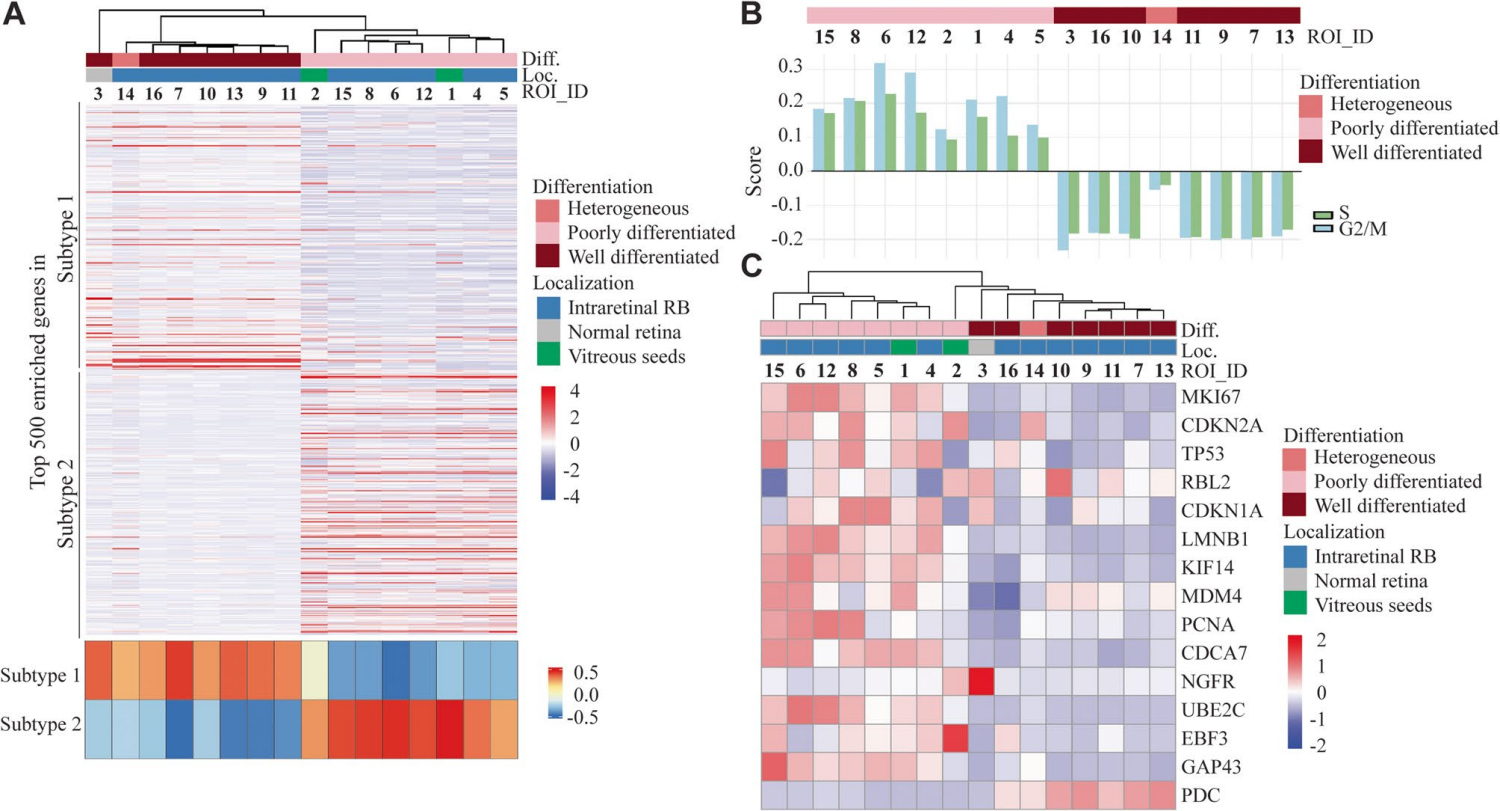
Molecular comparison of our ROIs with retinoblastoma subtypes/retinocytoma. **A** Top panel: Unsupervised clustering of the 16 ROIs based on subtype 1 and subtype 2 retinoblastoma signatures (top 500 upregulated genes in each subtype [11]). Bottom panel: Single-sample GSVA performed on RNA‐seq expression profiles of the 16 ROIs using gene‐set signatures defining subtype 1 and subtype 2. The heatmap displays GSVA‐derived enrichment scores (red, positive; blue, negative) for each subtype (rows) across ROIs (columns), ordered according to the top panel. **B** Prediction of the cell cycle phase calculated for each ROI. ROIs are ordered along the x-axis to match the sample sequence shown in Fig. 2B. **C** Unsupervised clustering of the 16 ROIs and heatmap expression of cherry-picked genes, markers of senescence or retinocytoma/retinoblastoma distinction

To deepen the comparison with the previously described subtypes 1 and 2 retinoblastoma [11], the 16 ROIs were clustered based on the transcriptomic signature of each subtype extracted from Liu et al. [11] (top 500 upregulated genes of each subtype) (Fig. 4A). The ROI segregation was in line with the histological observation and molecular profiling. The GSVA (gene set variation analysis) scores, calculated for each ROI, clearly highlighted that WD cells were identified as a subtype 1 retinoblastoma and PD cells as a subtype 2 tumor. To our knowledge, subtype 1 and 2 gene signatures have not been specifically attributed to date to WD/retinocytoma and PD areas of retinoblastoma, respectively.

To further gain insights into the proliferative state of the WD versus PD areas, a prediction of the cell cycle phase was calculated for each ROI. Interestingly, in all PD ROIs there was a positive score for the S and G2M phases, indicating a high proliferative potential. At the same time, all WD ROIs, including the non-tumoral retina and the transition area, presented a negative score for the S and G2M phases, suggesting that on average the cells in these areas were in G1 or G0 phases (Fig. 4B). Further assessment of the proliferation index KI67 at both RNA and protein levels revealed that non-proliferative cells in the WD areas were surrounded by highly proliferative cells in the PD areas (Figs. 3 and 4C).

As retinocytoma has been previously described to be in a senescent state, we explored our transcriptomic data to better understand the involvement of senescence in the WD area and clarify the status of the WD area between retinocytoma and non-proliferative retinoblastoma subtype 1.

In association with the downregulation of pRB expression [6], senescence markers such as *p16*,* p130* (*RBL2*) and *p21*^*CIP1*^ (*CDKN1A*) are suspected to be upregulated while *LMNB1* downregulated in retinocytomas [7, 15]. Many other genes have been described as retinocytoma markers, such as *KIF14*, *MDM4* [16], *PCNA*, *CDCA7* [17] and *p75*^*NTR*^ (*NGFR*) [18]. Our results demonstrated that none of the retinocytoma markers were regulated as expected in the WD area compared to the PD area, except for *LMNB1*, partially *p130* (*RBL2*), and *MDM4 (*Fig. 4C*)*. None of the ROIs actually meets both histological and so far described molecular criteria for retinocytoma. Interestingly, the level of expression of *UBE2C* [8], *EBF3*, *GAP43*, and *PDC* [11] was reminiscent of a profile of non-proliferative subtype 1 retinoblastoma (cluster 1 population [11]), also cited in previous studies as cone precursors [8] and cone precursor-like [9].

## Discussion

While regular bulk transcriptomic analysis represents the average expression of the different cell populations within a sample, with an overrepresentation of the major population, spatial transcriptomics provides a high-resolution mapping of sample heterogeneity. Although it does not achieve single-cell resolution, our approach allows the analysis of regions of interest of about 50 cells each, facilitating the study of distinct regions observed histologically. Our results demonstrate, for the first time, a clear correlation between the histological features and the transcriptional subpopulations, allowing a better delineation of the cellular and molecular heterogeneity inherent in this tumor. Identifying these adjacent regions as retinoblastoma subtypes 1 and 2 allowed us to map the spatial organization of these 2 subtypes directly within the patient’s tumor. We observed that the 2 subtypes were localized in adjacent distinct regions, and were not homogeneously mixed within the tumor tissue. While this single case study needs to be strengthened by other cases, this raises several questions about the origin of growth and the evolution of each subtype. It may be asked whether the cell of origin is the same for these 2 subtypes and, if so, which one gave rise to the other. In their recent work, Liu et al. proposed 2 main non-exclusive hypotheses to explain the existence of 2 retinoblastoma subtypes [11]. The first one would be a spatially or temporally different cone of origin of the two subtypes (e.g. localized in the central versus the peripheral retina, or at different stages of maturation). The second one, more likely based on their detailed analysis of one case, would result from a dedifferentiation process of the subtype 1 retinoblastoma, giving rise to a subtype 2. Our observation of both subtypes in the same tumor, in a patient without a germline mutation, would tend to favor the second hypothesis, but only a system of cell tracking over time could definitively confirm this hypothesis. One can also wonder what the evolution of these two distinct regions could be and, looking at the proliferation index, we could easily anticipate that subtype 2 would have continued to outgrow subtype 1. This would emphasize the need for analytical techniques that reflect the heterogeneity of the tumor so as not to be misleading in the representation of subtypes.

In addition, our single case report highlights the molecular similarity between non-proliferative subtype 1 retinoblastoma and retinocytoma. Although neither is a proliferative tumor, the former is malignant while the latter is considered benign. While retinocytoma has mainly been described as a transient precursor of retinoblastoma, it might be considered whether a proliferating subtype 1 retinoblastoma could evolve into a senescent retinocytoma. Our data describe an intermediate state with a non-proliferating subtype 1 retinoblastoma that does not show any of the typical senescent retinocytoma markers, although our ROI sampling could have missed an area of retinocytoma within the WD area. Whether this tumor state, which has been described in several studies, evolves into a retinocytoma or a proliferative subtype 1 retinoblastoma is a key point in correctly anticipating tumor evolution.

We acknowledge that a limitation of our study relies in the inclusion of only a single case and the analysis of further cases with adjacent well and poorly differentiated areas would be required to confirm our findings. Unraveling the molecular characteristics and spatiotemporal evolution of these different tumor types will provide knowledge that will allow better patient care. As liquid biopsies emerge as a potential tool for accessing tumors, it is essential to identify detectable biomarkers in ocular fluids that reflect all tumor forms in order to provide better treatment guidelines.

Furthermore, a deeper understanding of the intrinsic mechanisms driving the heterogeneity of retinoblastoma could benefit all the other pRB-loss tumors. The genetically stable context of retinoblastoma compared to other tumor types [19] facilitates its analysis and the discovery of common mechanisms.

## Conclusions

Our study reveals the spatial co-existence of subtype 1 and subtype 2 retinoblastoma within the same tumor and demonstrates a clear correlation between the histological and molecular features of the two subtypes. In addition, we could point out the molecular proximity between a non-proliferative subtype 1 retinoblastoma and a retinocytoma. This work, which needs to be further confirmed in a larger cohort of samples, provides key elements to better decipher the mechanisms of evolution of pRB-loss tumors.

## Supplementary Information

Additional file 1: Additional Figure 1. Study of the immune microenvironment of the tumor. A ESTIMATE-derived tumor purity and immune infiltration across spatial ROIs Stacked bar-plot showing the percentage of tumor (grey) versus non‐tumor/immune (red) cells in each of the 16 ROIs, as computed by the ESTIMATE algorithm on RNA‐seq expression profiles. ROIs are ordered along the x-axis to match the sample sequence shown in Fig. 2B. B EPIC‐inferred stromal and immune cell composition across spatial ROIs. Stacked bar‐plot showing the percent of all cells in each of the 16 ROIs attributed to seven major cell populations by EPIC deconvolution of RNA-seq data. Each bar is subdivided into natural killer (NK) cells (brown), macrophages (yellow), endothelial cells (orange), CD8⁺ T-cells (purple), CD4⁺ T-cells (green), cancer-associated fibroblasts (CAFs, blue), and B-cells (red). ROIs are ordered along the x-axis in the same sequence as in Fig. 2B. C Boxplots of ESTIMATE-derived tumor purity (%) in well- versus poorly-differentiated ROIs. Each point represents one ROI, with the box spanning the interquartile range and the horizontal line at the median. A two-sided Wilcoxon rank-sum test was used to compare the groups (p = 0.87). D Boxplots display the percentage of B cells, CD4⁺ T cells, CD8⁺ T cells, macrophages, and NK cells in well-differentiated or poorly-differentiated grouped ROIs. Individual ROI values are overlaid as points. Two-sided Wilcoxon rank-sum tests were performed for each cell type (ns = p > 0.05).

## Data Availability

The datasets used and/or analyzed during the current study are available from the corresponding author on reasonable request.

## Abbreviations

RB1: Retinoblastoma 1 gene
pRB: protein Retinoblastoma 1
scRNA-seq: single-cell RNA sequencing
FFPE: Formalin-Fixed Paraffin-Embedded
DSP: Digital Spatial Profiling
PBS: Phosphate-Buffered Saline
SSC: Saline Sodium Citrate
ROI: Region Of Interest
DEPC: Diethylpyrocarbonate
NGS: Next Generation Sequencing
PCR: Polymerase Chain Reaction
DEG: Differentially Expressed Genes
GSEA: Gene Set Enrichment Analysis
KEGG: Kyoto Encyclopedia of Genes and Genomes
GSVA: Gene Set Variation Analysis
PD: Poorly Differentiated
WD: Well Differentiated
tSNE: t-distributed Stochastic Neighbor Embedding

## References

[1] Knudson AG. Jr. Mutation and cancer: statistical study of retinoblastoma. Proc Natl Acad Sci U S A. 1971;68(4):820–3. 5279523 10.1073/pnas.68.4.820 PMC389051

[2] Munier FL, Mosimann P, Puccinelli F, Gaillard MC, Stathopoulos C, Houghton S, et al. First-line intra-arterial versus intravenous chemotherapy in unilateral sporadic group D retinoblastoma: evidence of better visual outcomes, ocular survival and shorter time to success with intra-arterial delivery from retrospective review of 20 years of treatment. Br J Ophthalmol. 2017;101(8):1086–93. 27927678 10.1136/bjophthalmol-2016-309298 PMC5537510

[3] Munier FL, Gaillard MC, Balmer A, Soliman S, Podilsky G, Moulin AP, et al. Intravitreal chemotherapy for vitreous disease in retinoblastoma revisited: from prohibition to conditional indications. Br J Ophthalmol. 2012;96(8):1078–83. 22694968 10.1136/bjophthalmol-2011-301450

[4] Munier FL, Moulin A, Gaillard MC, Bongiovanni M, Decembrini S, Houghton S, et al. Intracameral chemotherapy for globe salvage in retinoblastoma with secondary anterior chamber invasion. Ophthalmology. 2018;125(4):615–7. 29208450 10.1016/j.ophtha.2017.11.010

[5] Munier FL, Beck-Popovic M, Chantada GL, Cobrinik D, Kivela TT, Lohmann D, et al. Conservative management of retinoblastoma: challenging orthodoxy without compromising the state of metastatic grace. Alive, with good vision and no comorbidity. Prog Retin Eye Res. 2019. 10.1016/j.preteyeres.2019.05.005. 31173880 10.1016/j.preteyeres.2019.05.005

[6] Singh HP, Wang S, Stachelek K, Lee S, Reid MW, Thornton ME, et al. Developmental stage-specific proliferation and retinoblastoma genesis in RB-deficient human but not mouse cone precursors. Proc Natl Acad Sci U S A. 2018;115(40):E9391-400. 30213853 10.1073/pnas.1808903115 PMC6176579

[7] Dimaras H, Khetan V, Halliday W, Orlic M, Prigoda NL, Piovesan B, et al. Loss of RB1 induces non-proliferative retinoma: increasing genomic instability correlates with progression to retinoblastoma. Hum Mol Genet. 2008;17(10):1363–72. 18211953 10.1093/hmg/ddn024

[8] Yang J, Li Y, Han Y, Feng Y, Zhou M, Zong C, et al. Single-cell transcriptome profiling reveals intratumoural heterogeneity and malignant progression in retinoblastoma. Cell Death Dis. 2021;12(12): 1100. 34815392 10.1038/s41419-021-04390-4 PMC8611004

[9] Wu C, Yang J, Xiao W, Jiang Z, Chen S, Guo D, et al. Single-cell characterization of malignant phenotypes and microenvironment alteration in retinoblastoma. Cell Death Dis. 2022;13(5): 438. 35523772 10.1038/s41419-022-04904-8 PMC9076657

[10] Collin J, Queen R, Zerti D, Steel DH, Bowen C, Parulekar M, et al. Dissecting the transcriptional and chromatin accessibility heterogeneity of proliferating cone precursors in human retinoblastoma tumors by single cell sequencing-opening pathways to new therapeutic strategies. Invest Ophthalmol Vis Sci. 2021;62(6): 18. 34003213 10.1167/iovs.62.6.18 PMC8132003

[11] Liu J, Ottaviani D, Sefta M, Desbrousses C, Chapeaublanc E, Aschero R, et al. A high-risk retinoblastoma subtype with stemness features, dedifferentiated cone states and neuronal/ganglion cell gene expression. Nat Commun. 2021;12(1): 5578. 34552068 10.1038/s41467-021-25792-0 PMC8458383

[12] Yoshihara K, Shahmoradgoli M, Martinez E, Vegesna R, Kim H, Torres-Garcia W, et al. Inferring tumour purity and stromal and immune cell admixture from expression data. Nat Commun. 2013;4: 2612. 24113773 10.1038/ncomms3612 PMC3826632

[13] Racle J, Gfeller D. EPIC: A tool to estimate the proportions of different cell types from bulk gene expression data. Methods Mol Biol. 2020;2120:233–48. 32124324 10.1007/978-1-0716-0327-7_17

[14] Aschero R, Ganiewich D, Lamas G, Restrepo-Perdomo CA, Ottaviani D, Zugbi S, et al. Immunohistochemical expression of TFF1 is a marker of poor prognosis in retinoblastoma. Pediatr Blood Cancer. 2024;71(1): e30717. 37814421 10.1002/pbc.30717

[15] Zhang L, Pitcher LE, Yousefzadeh MJ, Niedernhofer LJ, Robbins PD, Zhu Y. Cellular senescence: a key therapeutic target in aging and diseases. J Clin Invest. 2022. 10.1172/JCI158450. 35912854 10.1172/JCI158450 PMC9337830

[16] Theriault BL, Dimaras H, Gallie BL, Corson TW. The genomic landscape of retinoblastoma: a review. Clin Exp Ophthalmol. 2014;42(1):33–52. 24433356 10.1111/ceo.12132 PMC3896868

[17] Liu H, Zhang Y, Zhang YY, Li YP, Hua ZQ, Zhang CJ, et al. Human embryonic stem cell-derived organoid retinoblastoma reveals a cancerous origin. Proc Natl Acad Sci U S A. 2020;117(52):33628–38. 33318192 10.1073/pnas.2011780117 PMC7776986

[18] Dimaras H, Coburn B, Pajovic S, Gallie BL. Loss of p75 neurotrophin receptor expression accompanies malignant progression to human and murine retinoblastoma. Mol Carcinog. 2006;45(5):333–43. 16555252 10.1002/mc.20179

[19] Kooi IE, Mol BM, Massink MP, Ameziane N, Meijers-Heijboer H, Dommering CJ, et al. Somatic genomic alterations in retinoblastoma beyond *RB1* are rare and limited to copy number changes. Sci Rep. 2016;6: 25264. 27126562 10.1038/srep25264 PMC4850475

