# Supplementary figures and images for: Spatial transcriptomics of retinoblastoma: a visual window on intra-patient heterogeneity
Source: BMC Cancer. 2025 Sep 2;25:1410. doi: 10.1186/s12885-025-14814-5 (PMC12403291; doi:10.1186/s12885-025-14814-5)

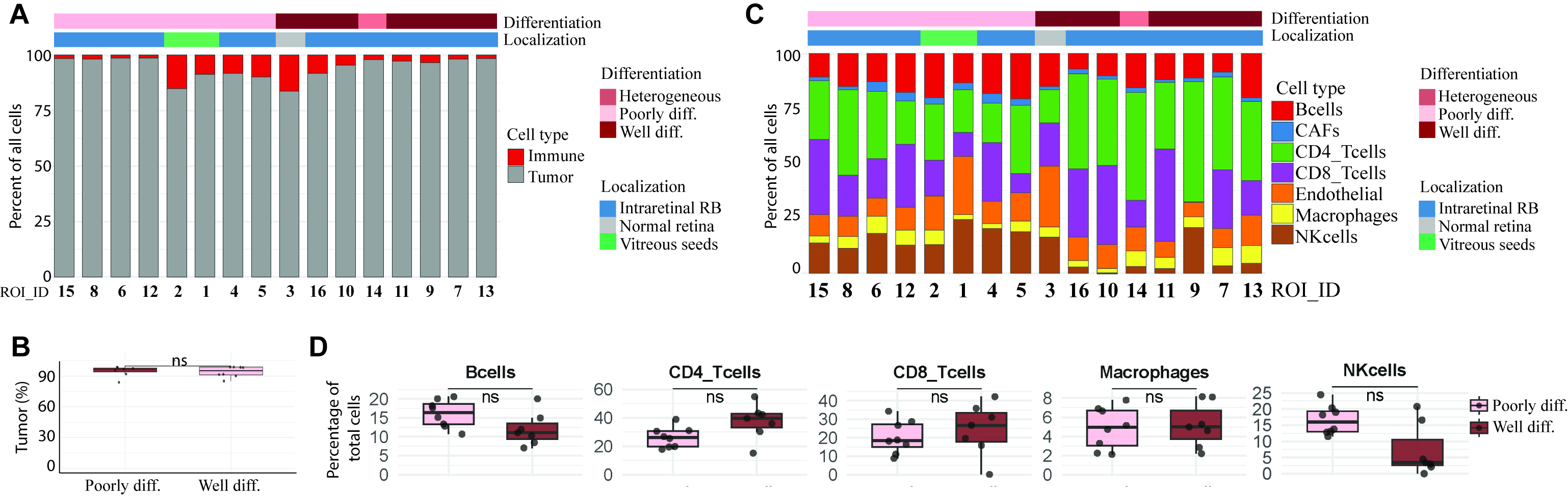

Supplement: Supplementary file 1 — Additional file 1: Additional Figure 1. Study of the immune microenvironment of the tumor. A ESTIMATE-derived tumor purity and immune infiltration across spatial ROIs Stacked bar-plot showing the percentage of tumor (grey) versus non‐tumor/immune (red) cells in each of the 16 ROIs, as computed by the ESTIMATE algorithm on RNA‐seq expression profiles. ROIs are ordered along the x-axis to match the sample sequence shown in Fig. 2B. B EPIC‐inferred stromal and immune cell composition across spatial ROIs. Stacked bar‐plot showing the percent of all cells in each of the 16 ROIs attributed to seven major cell populations by EPIC deconvolution of RNA-seq data. Each bar is subdivided into natural killer (NK) cells (brown), macrophages (yellow), endothelial cells (orange), CD8⁺ T-cells (purple), CD4⁺ T-cells (green), cancer-associated fibroblasts (CAFs, blue), and B-cells (red). ROIs are ordered along the x-axis in the same sequence as in Fig. 2B. C Boxplots of ESTIMATE-derived tumor purity (%) in well- versus poorly-differentiated ROIs. Each point represents one ROI, with the box spanning the interquartile range and the horizontal line at the median. A two-sided Wilcoxon rank-sum test was used to compare the groups (p = 0.87). D Boxplots display the percentage of B cells, CD4⁺ T cells, CD8⁺ T cells, macrophages, and NK cells in well-differentiated or poorly-differentiated grouped ROIs. Individual ROI values are overlaid as points. Two-sided Wilcoxon rank-sum tests were performed for each cell type (ns = p > 0.05). [file 12885_2025_14814_MOESM1_ESM.jpg]
